# Ultrasound-guided intra-tumoral administration of directly-injected therapies: a review of the technical and logistical considerations

**DOI:** 10.1186/s40644-024-00763-y

**Published:** 2024-10-25

**Authors:** George Gabriel Bitar, Melissa Persad, Alina Dragan, Adebayo Alade, Pablo Jiménez-Labaig, Edward Johnston, Samuel J Withey, Nicos Fotiadis, Kevin J. Harrington, Derfel ap Dafydd

**Affiliations:** 1 The Royal Marsden NHS Foundation Trust, Fulham Road, London, UK; 2 St George’s NHS foundation trust, London, UK; 3 The Institute of Cancer Research, Fulham Road, London, UK

**Keywords:** Ultrasound, CT, Directly-injected therapies, Head and neck, Oncology

## Abstract

**Background:**

Directly-injected therapies (DIT) include a broad range of agents within a developing research field in cancer immunotherapy, with encouraging clinical trial results in various tumour subtypes. Currently, the majority of such therapies are only available within clinical trials; however, more recently, talimogene laherparepvec (T-VEC, Imlygic) has been approved as the first oncolytic virus therapy in the USA and Europe. Our institution contributes to multiple different trials exploring the efficacy of DIT, the majority of which are performed by oncologists in clinic. However, specific, challenging cases – mainly neck tumours – require image-guided administration.

**Main body:**

This review article addresses the technical and logistical factors relevant to the incorporation of image-guided DIT into an established ultrasound service. Image-guidance (usually with ultrasound) is frequently needed for certain targets that cannot be palpated or are in high-risk locations, e.g. adjacent to blood vessels. A multi-disciplinary approach is essential to facilitate a safe and efficient service, including careful case-selection. Certain protocols and guidance need to be followed when incorporating such a service into an established ultrasound practice to enhance efficiency and optimise safety. Key learning points are drawn from the literature and from our early experience at a tertiary cancer centre following image guided DIT for an initial cohort of 22 patients (including 11 with a neck mass), addressing trial protocols, pre-procedure work-up, organisation, planning, consent, technical aspects, procedure tolerability, technical success, and post-procedure considerations.

**Conclusion:**

With appropriate planning and coordination, and application of the learning points discussed herein, image-guided administration of DIT can be safely and efficiently incorporated into an established procedural ultrasound list. This has relevance to cancer centres, radiology departments, individual radiologists, and other team members with a future role in meeting the emerging need for these procedures. This paper provides advice on developing such an imaging service, and offers certain insights into the evolving remit of radiologists within cancer care in the near future.

## Background

Directly-injected therapies (DIT) comprise a developing research field in cancer immunotherapy with encouraging clinical trial results in several tumour types, including head and neck squamous cell carcinoma (HNSCC), among other tumour types. DIT comprise a broad range of therapies including the direct administration of immunotherapy, modified oncolytic viruses and peptides, bacteria and chemotherapies.

Cancer cells have developed strategies to evade the immune system which allows them to thrive undetected within the host [1, 2]. Viruses, including herpes simplex virus and adenovirus, can be genetically modified selectively to target and kill tumour cells without harming normal cells [3, 4]. Immunotherapy works by priming the immune response against tumour-associated antigens so that they are recognised and targeted by host cells. The clinical goals of DIT include an objective response at the treated site by virtue of a higher concentration of drug in the target tumour. A further goal is an attenuated systemic adverse event profile that includes irreversible endocrine dysfunction (e.g. pancreatic insufficiency, hypothyroidism), colitis, hepatitis or pneumonitis [5, 6]. Injection of the enestic (target) lesion may produce immunologic effects at distant sites in nonenestic (uninjected) lesions i.e. abscopal effects [7–10]. Other goals include the treatment of sanctuary sites (where it is difficult to get a sufficient concentration of chemotherapy or infiltration of immune cells e.g. bones and ovaries) provision of symptom relief and downsizing and reducing the recurrence of tumours in the neo-adjuvant setting [6]. Whilst the majority of DIT are done in clinic by oncologists without image guidance, a select number of cases will be referred to radiology for injection using image guidance, usually with ultrasound following administration of local anaesthetic.

## Aims

Our institution, which has a large head and neck practice, takes part in several trials investigating intratumoral injection of immunotherapy, oncolytic viruses and other agents. Based on our experience to date, the aim of this paper is to provide: (a) a technical overview of how to safely perform ultrasound-guided injection of oncolytic viruses/ intratumoral immunotherapy; and (b) to describe the logistical aspects of incorporating such a service into existing ultrasound lists.

## Case selection and planning

Case planning involves co-ordination with the multidisciplinary team including oncologists, nurse specialists, research nurses, pharmacists and radiologists (including interventional radiology). Several key considerations for case planning will be addressed.

### Case selection

Trial protocols vary and some may stipulate that ultrasound-guidance should be used in all cases, whereas others may have a more selective approach. Certain protocols may permit ‘free-hand’ (non-image guided) injection of tumours if they are superficial, visible (or easily palpable), and well clear of major vascular structures. The need for image guidance is decided at a multi-disciplinary meeting (MDT). Some trials inject both the primary site in the head and neck for e.g., hypopharynx or oropharynx (performed by head and neck surgeons endoscopically) and the nodal sites of disease. As part of clinical trials, eligible patients would have had baseline cross-sectional imaging (usually CT, with or without MR) which allows tumour characteristics to be assessed in terms of extent, presence of necrosis, local invasion and vascular encasement. Absolute contraindications (identifiable on imaging) include intracranial invasion and/or tumour encasing the carotid arteries (due to the risk of stroke or carotid blow-out), jugular veins or thoracic great vessels (including the braciocephalic and subclavian arteries and veins). Wenig et al. [11] have reported the occurrence of fatal complications with subsequent modification of trial protocols to exclude tumours directly invading or near the common and internal carotid arteries, with no additional treatment-related cerebrovascular effects.

Using ultrasound guidance confers several advantages. Firstly, targeting deeper lesions that cannot be palpated and for which target identification in the absence of ultrasound would be challenging. Secondly, within the context of necrotic tumours, ultrasound-guidance facilitates targeting of the solid component of the tumour which is likely to increase efficacy of treatment. Thirdly, ultrasound ensures that administration of the drug is performed safely when target lesions are near critical structures including blood vessels. Furthermore, ultrasound-guidance allows assessment of tumour volume and additional parameters, specifically the presence of necrosis (which may suggest treatment response) and doppler flow, and thus can provide a guide with respect to treatment response (although CT is classically used for RECIST, iRECIST and IT RECIST measurement). Regions well suited to US guided injection include the head and neck, scalp, pleura, limbs, and superficial masses in the torso. Generally, tumours located deeper in the torso, for example in the lungs or deep to the peritoneum, require CT guided injection.

### Pre-procedure work-up and consent

Some trial protocols will require participants to have pre-procedure blood tests (including full blood count and International Normalised Ratio). Baseline cross-sectional imaging is mandatory and would normally include a CT of the neck, chest and abdomen. MRI may be used in certain cases, for example, to stage tumours that are poorly visualised or obscured by artefact (dental amalgam). Informed consent would need to be obtained from the patient for participation in a trial and, specifically, for the potential complications of the intratumoral injection, which vary in scope, frequency and severity depending on the drug(s). The patient will need to be provided with information about the nature of the trial, drug-specific complications (which should be provided by the pharmaceutical companies), the requirements for further injections and imaging follow-up. Clinical and research nurse specialists have an important role in supporting patients throughout the process, providing information and addressing any concerns that patients may have.

### Organisation and planning

Close collaboration with both pharmacy and the research team is important because intratumoral drug comes pre-prepared and delivery can be time-sensitive, such that delays may lead to expiry of the investigational medical product. Most of the drugs have a short time to expiry so are prepared on the day at the pharmacy. In some cases, such as with the drug IXOVEX (an adenovirus), the time to expiry is only thirty minutes, and therefore the drug needs to be prepared in the ultrasound room where the injection is being performed. Availability of the drug needs to be confirmed prior to the patient’s appointment time to ensure that the drug is available, and an ultrasound slot is not wasted with the associated inconvenience caused to healthcare staff and patients. The dates and times of the treatment is pre-determined by trial protocol and, so, generally cannot be easily altered to meet clinician and/or patient preferences or fixed radiology lists, although allowances should be made where possible. Ideally, there should be at least two or three radiologists trained to perform the procedures who are familiar with the trial protocol. This allows for continuous service provision, including annual leave cover, and ensures that appointment times and adherence to trial protocols is maintained.

Ideally, a double ultrasound slot (at our institution, typically fifteen minutes for a diagnostic and thirty minutes for a procedural case) should be booked as this allows time to discuss and formally consent the patient for the procedure, as well as adequate time to perform the injection without haste. It is preferable to have the slot at the end of an ultrasound list to minimise contamination of the local environment by spillage or aerosolization of the injectate.

### Pre-procedural preparation

Patients generally prefer a small team of familiar operators. Given that DIT are infrequently performed and currently only as part of a clinical trial, it is preferable to both patients and medical staff that a small team of familiar operators well-versed in trial protocols and technical procedural aspects performs the procedure. These operators should receive formal training of “Hazardous drug handling” as some of the drugs used e.g. Tiganol Tigate are highly irritant. At our institution, a group of three operators perform these injections on a regular basis and are named on trial delegation logs. Other members of the research team who are highly familiar with DIT and their particulars are also routinely present during the procedures, adding a further level of oversight and safety.

Suitable personal protective equipment, specifically gloves, a gown/apron and safety goggles need to be available. Sometimes, trial protocols require that patients have immediate pre- and post-procedure bloods, electrocardiography, and monitoring of blood pressure and other vital signs before and during the injection. Rarely, sedation with midazolam may be required, particularly if the patient is anxious or multiple prior attempts have been performed that have resulted in local inflammation and/or discomfort. If the patient is having radiotherapy treatment, there will need to be consideration of the timing of DIT with respect to radiotherapy planning. Ideally, DIT should be administered before or during the first few weeks of radiotherapy due to increased risks of radiodermatitis and mucositis in the second half of radiotherapy which may increase the risk of ulceration or infection.

Before each injection, an assessment with ultrasound may be performed to assess the target lesion(s), to obtain measurements (length, width and height [cm] and volume [cm^3^]) and evaluate for any treatment response (if injections have been previously performed). This assessment will inform the operator as to the required dose and volume of drug to be injected, as this should be adjusted if the shrinks. Pertinent data, as in Table 1, is recorded for trial purposes. Where multiple tumours are present, the preferred injection target is agreed between the research team and radiologist; whichever target is regarded as the safest, technically easiest, and largest tumour (in that order of priority). Ideally, measurements should be obtained in three perpendicular dimensions ensuring that the maximum dimension in any plane is recorded. However, response evaluation will usually be better assessed on contrast enhanced CT which enables more objective measurements and staging of all lesions, an assessment for abscopal effects and application of the Response Evaluation Criteria in Solid Tumours version 1.1 (RECIST1.1) [12–14]. Identification/land-marking the injection target can be important with clustered/ multifocal disease where the target may be hard to discern and to ensure that the same lesion is injected on subsequent visits (if that is stipulated per protocol). Of note, if there is a response to treatment, the target lesion can become harder to measure or to inject on subsequent visits due to tumour shrinkage.

**Table 1:** Example of a reporting template and data recorded for trial purposes

Target tumour side, location and texture e.g. right lateral neck with central necrosis	
Current [modality] measurements e.g. 30 (AP) x 35 (LR) x 40 (CC) mm	
Informed written consent for procedure previously performed by clinical team. Verbal consent obtained immediately prior to the procedure	
WHO checklist completed	
Aseptic conditions	
Local anaesthesia to skin and subcutaneous soft tissues e.g. 5mL of 1% lidocaine	
Procedure conducted under ultrasound guidance	
Pre-prepared injectate (see patient notes for dosage/volume) distributed into the solid, peripheral components of the target tumour in a single pass	
No immediate complications	
Standard post-procedure care according to trial protocol	

### Technical procedural aspects

**Fig. 1:**
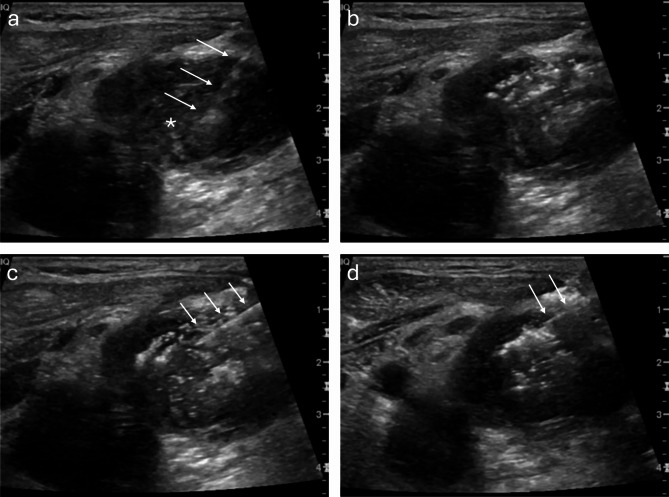
B-mode ultrasound images of a neck tumour (*) targeted for DIT. (**a**) Initial pass with needle into the deeper aspect of the target lesion. (**b**) hyperechoic gas bubbles on injection from pre-prepared injectate. (**c**, **d**) Superficial passes into the target lesion

A WHO checklist should be completed, and aseptic technique observed for all procedures. Once the target lesion has been identified, the most appropriate approach will need to be considered and the patient positioning optimised. Ideally, as with any ultrasound-guided procedure, the radiologist, probe and the ultrasound screen should be arranged to make the procedure comfortable and easier to perform for the radiologist which, in turn, will ensure optimal delivery of the injectate. In most cases, local anaesthetic is administered from the skin surface to the tumour edge (5 ml, 1% lignocaine); however, this may not always be required, for example if the target is easily accessible or if the patient can tolerate the procedure and is reluctant to have local anaesthesia. The radiologist is provided with a syringe (typically 5 ml, Leur lock), containing the pre-dosed, pre-prepared injectate, as supplied by pharmacy. Prior to administration, it is important to ensure that no residual air is expelled from the syringe containing the pre-prepared injectate as this could turn the injectate (containing genetically modified microorganisms) into an aerosol. The volume/dose of injectate is predetermined by the research team and pharmacy, and will be governed by parameters including patient size and tumour size. The needle (typically 23 Gauge [blue]) should initially be directed into the deeper aspect of the target lesion, and this aspect injected first, as in Fig. 1, before the more superficial components of the lesion are injected (usually into four quadrants of the tumour, if feasible). This ensures that any initially expelled gas does not obscure the deeper parts of the lesion which could be problematic if the more superficial components of the lesion were injected first. The edges and peripheries of the lesion will need to be injected due to higher immune cell accessibility as the periphery of the tumour often has a higher density of blood vessels compared to its core. This is particularly relevant in the case of large centrally necrotic tumours where the solid and vascular elements of the tumour that are generally at the periphery are targeted at several (up to four) points. This increased vascularity allows better access for the immune cells and therapeutic agents, potentially enhancing the effectiveness of treatment. The periphery generally has a more oxygen rich environment than the core, which can support more active and effective immune responses. The core of a tumour often contains dense extracellular matrix and fibrotic tissue, which can physically prevent the penetration of the drug. Spillover of drug at the periphery of tumour and into healthy tissue may trigger a systemic immune response. These reasons make the periphery of the tumour a critical target for DIT, potentially increasing the overall efficacy of the immunotherapy.

The treated neck can be particularly challenging to address, as patients will often have had previous radiotherapy and surgery which results in fibrosis, scarring and a firm, sclerotic texture to the soft tissues. This may require some force to inject the treatment into the target lesion, and once the lesion is injected and the needle has been removed, the puncture site should be covered with the ultrasound probe to enable dispersion of the injectate in the target and avoid jets of injectate being expelled from the skin.

### Post-procedural considerations

Following the procedure, ultrasound should be performed to ensure that there is no haematoma that may be suggestive of vascular injury. The patient should be transferred to a designated clinical area for monitoring in an armchair (or daybed if available) for a pre-specified period (according to trial protocol) and provided with steroid cover to prevent severe inflammatory reactions and oral analgesia, if any pain develops as the local anaesthetic wears off. In some cases, the injections are painful enough to require oral or intravenous opioids for e.g., fentanyl. The ultrasound measurements of the target lesions, the technical aspects of the procedure and the presence or absence of any immediate complications should be clearly documented in a report on the radiology information system. The ultrasound room will need either a standard or a deep-clean depending on how hazardous the exact type of drug injected is.

### Directly-injected therapies: our local experience

The approach to image guided DIT described in this article was developed through our early experience at our local cancer centre. For context, in between January 2018 and January 2024, 71 patients have undergone DIT at our institution, in the context of 9 clinical trials. Of these, 21 cases (30%) were performed with ultrasound guidance, 11 (50%) of which were neck masses, with a median number of 7 injections performed under image-guidance (range 2–25), totalling more than 150 procedures.

The procedures were all well-tolerated and there were no immediate technical complications related to the needle insertion and injectate delivery. As several of the trials are ongoing, outcome data, including delayed systemic symptoms related to the oncolytic viral treatments will be published in due course.

**Fig. 2:**
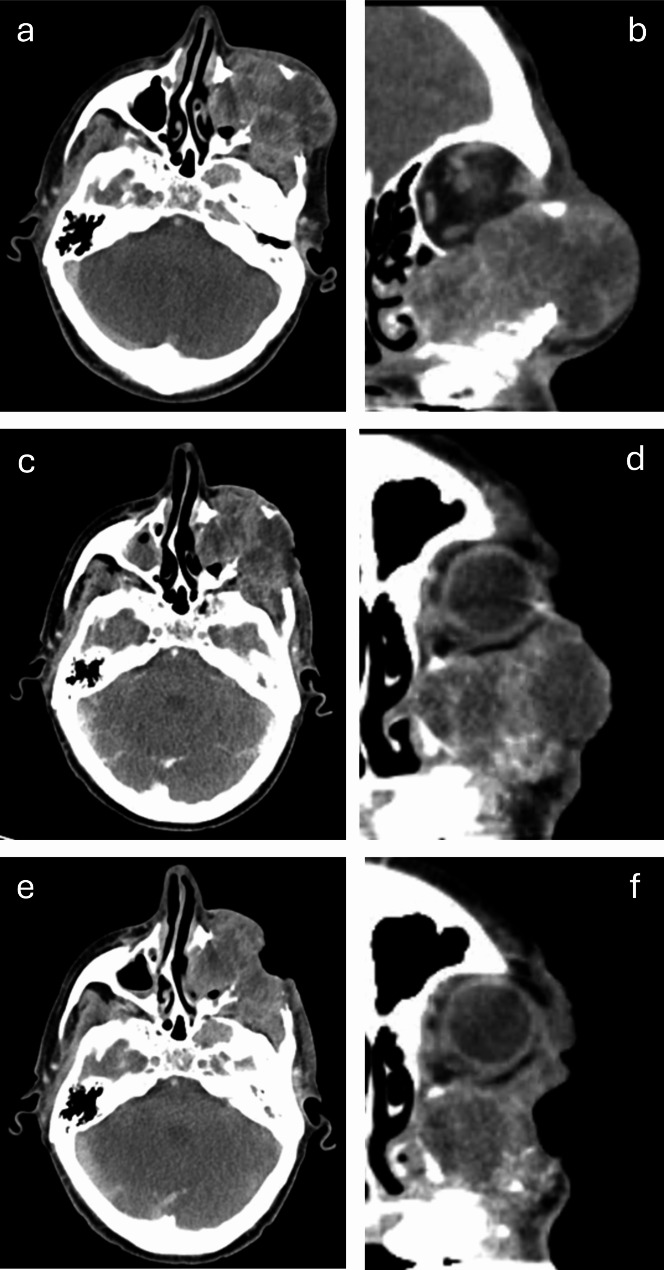
Axial and coronal contrast-enhanced CT images of locally advanced adenocarcinoma involving the left maxilla, mandible and orbit. (**a**, **b**) Recurrence several years after extended left maxillectomy, before DIT (**c**, **d**) tumour shrinkage 6 weeks after initial DIT session. (**e**, **f**) further reduction in size of the tumour 3 months after initial DIT session

**Fig. 3:**
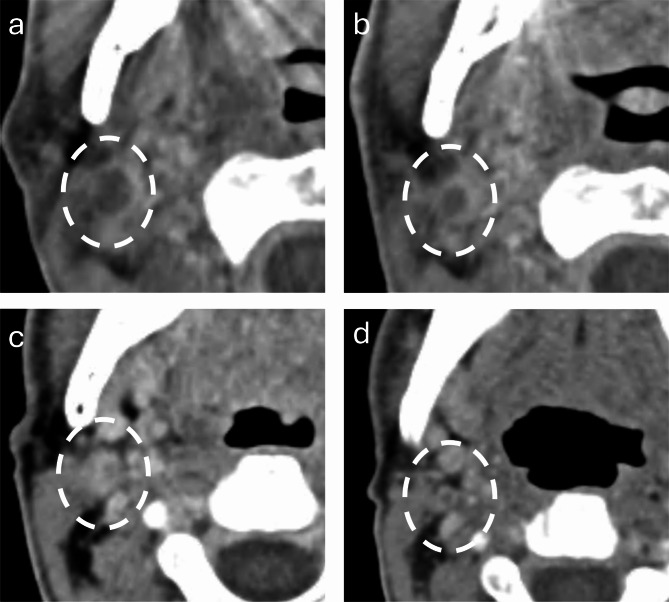
Axial contrast-enhanced CT images of a serially injected necrotic right level II lymph node related to a right parotid oncocytic carcinoma. (**a**) Baseline imaging one month prior to the first DIT session. (**b**) Reduction in size of the metastatic lymph node after four DIT sessions. (**c**, **d**) Ongoing reduction in size after three subsequent DIT sessions (12 months from baseline)

Figures 2 and 3 show sequential images of two patients treated for a maxillary adenocarcinoma and a parotid oncocytic carcinoma, respectively.

## Discussion

DIT represents a developing clinical and research field with some promising clinical trial results within the context of HNSCC and other malignancies. The first systematic review to evaluate DIT comprised 31 studies treating 948 patients with confirmed HNSCC, 11 (35%) of which added systemic therapy or radiotherapy [15]. The clinical trials investigated nineteen DIT treatments including chemotherapy, immunotherapy, nanoparticle and micro-organism-based agents. As noted by the authors, drawing generalisable conclusions is challenging due to various factors including that the outcome data of the individual clinical trials are not fully assessable and comparable across the studies. However, the overall response rate (ORR) achieved encouraging values greater than 40% and 60% in five and three studies, respectively. Whilst encouraging, the authors conclude that the magnitude of clinical benefit and the quality of the evidence available is insufficient to recommend the application of DIT as standard treatments outside of clinical trials.

DIT is only available in our institution as part of ongoing trials investigating their efficacy and safety; however, more recently talimogene laherparepvec (T-VEC), an oncolytic herpes simplex type 1 (HSV-1) virus has received Food and Drug Administration (FDA), European Medicines Agency (EMA) and National Institute of Clinical Excellence (NICE) approval for the treatment of unresectable melanoma [16–20]. Although the mechanism of action of T-VEC is not completely elucidated, it is thought to induce immunogenic cell death and activation of the host’s anti-tumoral immune response [20, 21]. A wide range of other DIT has been trialled within the context of recurrent HNSCC, including Poly-ICLC [22], cisplatin/epinephrine injectable gel [11], interleukin-2 therapy [23], mycobacterial heat-shock protein 65 [24], CAR-T [25] and microorganism-based IT treatments including oncolytic viruses [19, 26], plasmids [27, 28], and bacteria [29, 30]. At our institution, a variety of DIT are currently undergoing trial in patients with HNSCC, although results are not currently available. Although currently T-VEC is the only licensed DIT, it is probable that more treatments will be approved as trials exploring a wide breadth of potential uses conclude with positive outcome data.

To our knowledge, this is the first paper addressing the logistics of incorporating an image-guided DIT service into established procedural ultrasound lists. All tumour sites are potentially injectable, but the injection of some is more complicated than others and may require image guidance. Cases most suitable for ultrasound-guided DIT include lesions that cannot be palpated, have significant necrotic components (where the solid component should be targeted) or are near critical structures including blood vessels and the airways which should be avoided. Computed Tomography may be used for certain target lesions not visible or accessible with ultrasound e.g., retroperitoneal and intrapulmonary lesions. We have suggested how to incorporate image-guided DIT into an ultrasound service. We have focussed on adherence to trial protocols, involvement and co-ordination with various members of the multidisciplinary team, patient involvement, general set-up and considerations, co-ordination with pharmacy and, of course, safety aspects. A multidisciplinary team approach is essential to ensure a safe, efficient and seamless patient experience. This includes the involvement of clinicians, radiologists, pharmacists who provide the pre-prepared injectate (which needs to be ordered in advance) and research nurses. An overview of the more specific technical aspects of the procedure has also been provided to assist radiologists and interventional radiologists to perform procedures efficiently, safely and to ensure optimal delivery of the injectate into a target lesion.

From our early experience using this approach in over 150 image guided injections, the procedures were well-tolerated with no immediate complications, suggesting that DIT can be safely incorporated into an established ultrasound service. Jiménez-Labaig et al. also found in their systematic review that the most frequent severe or grade ≥3 treatment related adverse events was post-injection dysphagia in four trials, specifically in trials injecting cisplatin and epinephrine [15]. However, there were no common side effects across DIT beyond grade 1–2 pain at the injection site, local inflammation and potential cellulitis thereby affirming the tolerability of these treatments.

A further consideration is that whilst ultrasound-guided DIT is currently performed within a radiology department, point of care ultrasound (POCUS) may be a potential future avenue given the current workload pressures facing radiology departments. POCUS is ultrasound performed by clinicians or non-radiologists, for example in an outpatient setting. Depending on future demand for DIT, it may be appropriate at an early stage to encourage training of clinicians by radiologists in ultrasound guided DIT. This might apply for lower risk and lower complexity targets, whilst reserving capacity in radiology for higher risk, higher complexity targets.

DIT have the potential to significantly alter the future landscape of medical oncology and the role of radiologists in modern healthcare. Radiologists are already well-versed in image-guide procedures so the provision of a DIT service would constitute a natural extension of skills that, in principle, radiologists already have. Image-guided DIT might impact the work of radiologists undertaking image-guided instrumentation. It is hoped that the material covered in this paper will be of interest to specialists as they encounter the emerging demand for these procedures; this paper serving as a potential guide for radiologists and other team members with a role in future DIT related services.

## Data Availability

No datasets were generated or analysed during the current study.

## Abbreviations

DIT: Directly–Injected Therapies
TVEC: Talimogene Laherparepvec
HNSCC: Head and Neck Squamous Cell Carcinoma
MDT: Multi–disciplinary meeting
RECIST 1.1: Response Evaluation Criteria in Solid Tumours version 1.1
WHO: World Health Organisation
ORR: Overall Response Rate
FDA: Food and Drug Administration
EMA: European Medicines Agency
NICE: National Institute of Clinical Excellence

## References

[1] Messerschmidt JL, Prendergast GC, Messerschmidt GL. How cancers escape Immune Destruction and mechanisms of Action for the New significantly active Immune therapies: helping non-immunologists decipher recent advances. Oncologist. 2016;21(2):233–43. 26834161 10.1634/theoncologist.2015-0282 PMC4746082

[2] Mohme M, Riethdorf S, Pantel K. Circulating and disseminated tumour cells-mechanisms of immune surveillance and escape. Nat Rev Clin Oncol. 2017;143:155. 10.1038/nrclinonc.2016.144 27644321

[3] Lawler SE, Antonio Chiocca E. Oncolytic virus-mediated immunotherapy: a combinatorial Approach for Cancer Treatment. J Clin Oncol. 2015;33(25):2812–4. 26215964 10.1200/JCO.2015.62.5244

[4] Fukuhara H, Ino Y, Todo T. Oncolytic virus therapy: a new era of cancer treatment at dawn. Cancer Sci 2016 Oct:107(10):1373–9. 10.1111/cas.13027 PMC5084676 27486853

[5] Kichloo A, Albosta M, Dahiya D, et al. Systemic adverse effects and toxicities associated with immunotherapy: a review. World J Clin Oncol. 2021;12(3):150–63. 33767971 10.5306/wjco.v12.i3.150 PMC7968107

[6] Marabelle A, Andtbacka R, Harrington K, et al. Starting the fight in the tumor: expert recommendations for the development of human intratumoral therapy (HIT-IT). Ann Oncol. 2018;29(11):2163–74. 30295695 10.1093/annonc/mdy423 PMC6290929

[7] Tronnier M, Mitteldorf C. Treating advanced melanoma: current insights and opportunities. Cancer Manag Res. 2014;6:349–56. 25228821 10.2147/CMAR.S49494 PMC4164110

[8] Champiat S, Tselikas L, Farhane S, et al. Intratumoral Immunotherapy: from Trial Design to Clinical Practice. Clin Cancer Res. 2021;27(3):665–79. 10.1158/1078-0432.CCR-20-0473 32943460 10.1158/1078-0432.CCR-20-0473

[9] Marabelle A, Tselikas L, de Baere T, Houot R. Intratumoral immunotherapy: using the tumor as the remedy. Ann Oncol. 2017;28:xii33–43. 10.1093/annonc/mdx683 29253115 10.1093/annonc/mdx683

[10] Wang Z, Sun P, Li Z, Xiao S. Clinical advances and future directions of Oncolytic Virotherapy for Head and Neck Cancer. Cancers. 2023;15(21):5291. 10.3390/cancers15215291 37958464 10.3390/cancers15215291 PMC10650136

[11] Wenig BL, Werner JA, Castro D, et al. The role of Intratumoral Therapy with Cisplatin/Epinephrine Injectable Gel in the management of advanced squamous cell carcinoma of the Head and Neck. Arch Otolaryngol Head Neck Surg. 2002;128(8):880–5. 12162764 10.1001/archotol.128.8.880

[12] Eisenhauer EA, Therasse P, Bogaerts J, et al. New response evaluation criteria in solid tumours: revised RECIST guideline (version 1.1). Eur J Cancer. 2009;45(2):228–47. 19097774 10.1016/j.ejca.2008.10.026

[13] Seymour L, Bogaerts J, Perrone A, et al. iRECIST: guidelines for response criteria for use in trials testing immunotherapeutics. Lancet Oncol. 2017;18(3):e143–52. 28271869 10.1016/S1470-2045(17)30074-8 PMC5648544

[14] Goldmacher GV, Khilnani AD, Andtbacka RHI, et al. Response criteria for Intratumoral Immunotherapy in Solid tumors: itRECIST. J Clin Oncol. 2020;38(23):2667–76. 32552274 10.1200/JCO.19.02985 PMC7402995

[15] Jiménez-Labaig P, Rullan A, Braña I et al. Intratumoral therapies in head and neck squamous cell carcinoma: A systematic review and future perspectives. Cancer Treat Rev. 2024 Jun: 127:102746. 10.1016/j.ctrv.2024.102746 38696902

[16] Andtbacka RHI, Kaufman HL, Collichio F, et al. Talimogene Laherparepvec improves durable response rate in patients with Advanced Melanoma. J Clin Oncol. 2015;33(25):2780–8. 26014293 10.1200/JCO.2014.58.3377

[17] Harrington KJ, Michielin O, Malvehy J et al. A practical guide to the handling and administration of talimogene laherparepvec in Europe. Oncol Targets Ther 2017 Aug 2:10:3867–80. 10.2147/OTT.S133699 PMC5546812 28814886

[18] Andtbacka RHI, Collichio F, Harrington KJ, et al. Final analyses of OPTiM: a randomized phase III trial of talimogene laherparepvec versus granulocyte-macrophage colony-stimulating factor in unresectable stage III-IV melanoma. J Immunother Cancer. 2019;7(1):145. 31171039 10.1186/s40425-019-0623-z PMC6554874

[19] Harrington KJ, Hingorani M, Tanay MA, et al. Phase I/II study of Oncolytic HSVGM-CSF in Combination with Radiotherapy and Cisplatin in untreated stage III/IV squamous cell Cancer of the Head and Neck. Clin Cancer Res. 2010;16(15):4005–15. 20670951 10.1158/1078-0432.CCR-10-0196

[20] Harrington KJ, Kong A, Mach N, et al. Talimogene Laherparepvec and Pembrolizumab in recurrent or metastatic squamous cell carcinoma of the Head and Neck (MASTERKEY-232): a Multicenter, phase 1b study. Clin Cancer Res. 2020;26(19):5153–61. 32669371 10.1158/1078-0432.CCR-20-1170

[21] Bommareddy PK, Zloza A, Rabkin SD, et al. Oncolytic virus immunotherapy induces immunogenic cell death and overcomes STING deficiency in melanoma. Oncoimmunology. 2019;8(7):1591875. 31143509 10.1080/2162402X.2019.1591875 PMC6527276

[22] Kyi C, Roudko V, Sabado R et al. Therapeutic Immune Modulation against Solid Cancers with Intratumoral Poly-ICLC: a pilot trial. Clin Cancer Res 2018 Oct 15; 24(20): 4937–48. 10.1158/1078-0432.CCR-17-1866 10.1158/1078-0432.CCR-17-1866 PMC6191332 29950349

[23] Mattijssen V, De Mulder PH, De Graeff A, et al. Intratumoral PEG-interleukin-2 therapy in patients with locoregionally recurrent head and neck squamous-cell carcinoma. Ann Oncol. 1994;5(10):957–60. 7696170 10.1093/oxfordjournals.annonc.a058739

[24] Michaluart P, Abdallah KA, Lima FD, et al. Phase I trial of DNA-hsp65 immunotherapy for advanced squamous cell carcinoma of the head and neck. Cancer Gene Ther. 2008;15(10):676–84. 18535616 10.1038/cgt.2008.35

[25] Papa S, Adami A, Metoudi M, et al. Intratumoral pan-ErbB targeted CAR-T for head and neck squamous cell carcinoma: interim analysis of the T4 immunotherapy study. J Immunother Cancer. 2023;11(6):e007162. 37321663 10.1136/jitc-2023-007162 PMC10277526

[26] Hu JCC, Coffin RS, Davis CJ, et al. A phase I study of OncoVEXGM-CSF, a second-generation oncolytic herpes Simplex Virus expressing granulocyte macrophage colony-stimulating factor. Clin Cancer Res. 2006;12(22):6737–47. 17121894 10.1158/1078-0432.CCR-06-0759

[27] Clayman GL, el-Naggar AK, Lippman SM, et al. Adenovirus-mediated p53 gene transfer in patients with advanced recurrent head and neck squamous cell carcinoma. J Clin Oncol. 1998;16(6):2221–32. 9626224 10.1200/JCO.1998.16.6.2221

[28] Villaret D, Glisson B, Kenady D, et al. A multicenter phase II study of tgDCC-E1A for the intratumoral treatment of patients with recurrent head and neck squamous cell carcinoma. Head Neck. 2002;24(7):661–9. 12112540 10.1002/hed.10107

[29] Kitahara S, Ikeda M, Inouye T, et al. Inhibition of head and neck metastatic and/or recurrent cancer by local administration of multi-cytokine inducer OK-432. J Laryngol Otol. 1996;110(5):449–53. 8762314 10.1017/s0022215100133948

[30] Cheng VST, Suit HD, Wang CC, Raker J, Weymuller E, Kaufman S. A preliminary study of intralesional, intralymph node, intravenous and intraperitonealCorynebacterium parvum treatments in patients with advanced cancer. Cancer. 1978;42(4):1912–5. 709538 10.1002/1097-0142(197810)42:4<1912::aid-cncr2820420432>3.0.co;2-5

